# Barriers to responding to reproductive coercion and abuse among women presenting to Australian primary care

**DOI:** 10.1186/s12913-021-06420-5

**Published:** 2021-05-04

**Authors:** Molly Wellington, Kelsey Hegarty, Laura Tarzia

**Affiliations:** 1 Department of General Practice, The University of Melbourne, 780 Elizabeth Street, Melbourne, Vic Australia; 2 Centre for Family Violence Prevention, The Royal Women’s Hospital, Melbourne, Australia

## Abstract

**Background:**

Reproductive coercion and abuse is defined as any behaviour that seeks to control a woman’s reproductive autonomy. In Australia, women often access reproductive health care through a primary care clinician, however, little is known about clinicians’ experiences responding to reproductive coercion and abuse. This study aims to address this gap by exploring the barriers to responding to reproductive coercion and abuse in Australian primary care.

**Methods:**

In this qualitative study, twenty-four primary care clinicians from diverse clinical settings in primary care across Australia were recruited to participate in a semi-structured interview. Data were analysed thematically.

**Results:**

Through analysis, three themes were developed: *It’s not even in the frame*; which centred around clinicians lack of awareness around the issue. *There’s not much we can do,* where clinicians described a lack of confidence in responding correctly as well as a lack of services to refer on to. Lastly *There’s no one to help us,* explaining the disconnect between referral services and primary care as well as the impacts of lack of abortion on women experiencing reproductive coercion and abuse.

**Conclusions:**

Clinicians expressed similar experiences of barriers to respond to reproductive coercion and abuse. Many clinicians felt ill-equipped to identify and respond to reproductive coercion and abuse. Some clinicians hadn’t received any formal training, others were trained but had nowhere to refer women. Further complicating responses was a lack of support from referral services. This study highlights the need for more training and a streamlined referral pathways for women who experience reproductive coercion and abuse, as well as better access to reproductive health services in rural areas.

**Supplementary Information:**

The online version contains supplementary material available at 10.1186/s12913-021-06420-5.

## Background

Reproductive coercion and abuse (RCA) is a form of violence against women [1]. It is defined as any behaviour that seeks to control a woman’s reproductive autonomy, typically encompassing; tampering or removing access to contraception (contraceptive sabotage), threats or acts of violence to force a woman to become pregnant (pregnancy pressure or coercion) and forcing a woman to terminate a wanted pregnancy or continue an unwanted pregnancy (controlling the outcome of a pregnancy) [2]. Although most commonly labelled ‘reproductive coercion’ in literature and practice, recent research in the area has recommended the word ‘abuse’ be included since it highlights the intention and effect of the behaviours it describes [1, 3]. Male intimate, dating or ex-partners are often perpetrators of RCA, however, family members and other people can be responsible [4].

RCA shares commonalities with both intimate partner violence (IPV) and sexual violence (SV). Women who experience RCA are likely to co-experience IPV [2, 5], yet the nuances of this association are unclear. The relationships between physical, psychological and sexual IPV and RCA have not been explored to any great extent. This highlights an overall lack of knowledge about intersections between RCA and other forms of violence. Further, RCA is linked to negative physical and mental health outcomes both specific to RCA [6, 7] and more broadly in conjunction with IPV and SV [8]. Health services are an effective setting to identify and respond to IPV and SV more broadly [9, 10]. The World Health Organisation (WHO) has highlighted primary care as a health setting that is well placed to identify and respond to IPV and SV [11].

Primary care in Australia is universal access, diverse and complex consisting of all clinics having government subsidies per patient encounter, with some clinics charging additional fees for services [12]. Clinics can be large (more than three doctors) and also offer nursing and allied health services [13], or small with only one or two general practitioners (GPs) working with a single primary care nurse. Women generally access contraceptives and reproductive health services through primary care. To commence antenatal care, or access a termination of pregnancy, women need to see a GP to have their pregnancy confirmed, and some GPs can prescribe medical termination of pregnancy pills [14]. Additional barriers exist in the Australian health system around rural/remote settings and access to care such as termination of pregnancy and other reproductive health services [15, 16]. Women in rural/remote settings experience stigma and a lack of services that are more readily available to women in metropolitan areas. Given the reasons women access primary care, it is a setting where clinicians are likely to encounter women experiencing RCA.

Studies looking at RCA and health settings are scarce. An interview study (*n* = 17) into how clinicians understand RCA, showed that hospital doctors and nurses had varying opinions and understandings of what constitutes RCA and how it should be responded to [3]. Further, clinicians are aware of the issue and its impacts on their patients but believe that it is still mostly hidden and unacknowledged [3]. A lack of common understanding and the hidden nature of RCA in healthcare makes it difficult to implement interventions. Studies in the broader IPV context indicate that women trust clinicians and are willing to disclose experiences of violence to them, depending on the response given by the clinician [17]. Despite the trust given by women, clinicians in primary care have identified barriers to responding to IPV in practice, such as a lack of skills, training and time constraints [18]. In Australia, surveys have shown that GPs are professionals that women would chose to disclose IPV to, over other professionals such as justice workers or domestic/family violence workers [19].

Although GPs are trusted clinicians, very little is known about their experiences with RCA in their patients. One study (*n* = 17) conducted in a hospital have provided some insight into how RCA is responded to in a tertiary health setting [20], however there are no studies exploring primary care and the challenges they face in responding to RCA. In response to these gaps, this paper aims to qualitatively explore primary care clinicians’ perceptions of the barriers to responding to RCA in Australia. A qualitative method was chosen as there is scarce research in the area overall and using qualitative methods would allow for a more exploratory approach, giving participants the opportunity to explain nuances of their experiences. In particular, we focus on the barriers that are unique to RCA as a distinct form of violence against women.

## Method

### Study setting

This study was conducted in primary care settings across Australia. Funding and legislation around healthcare are often controlled by individual states rather than Federally [21]; this means that there can be large differences across the country when it comes to access to services and amount of funding put into primary care.

### Participant recruitment

We aimed to recruit participants from multiple states and varying degrees of rurality to ensure diversity. To achieve this, three different stages of recruitment were completed.

Inclusion criteria included doctors (general practitioners and specialists) who perform or prescribe termination of pregnancy medication and nurses who assist in this process.

The first method involved creating a partnership with a university regional sexual health centre who advertised the project through various channels including newsletters and clinical network meetings. Expression of interest advertisements were posted on key primary care social media sites, all authors posted advertisements through additional social media channels. Every primary health network (PHN) in Australia was contacted by the researchers to seek advice and assistance in recruitment, several PHN’s posted small advertisements in their monthly newsletters and on websites. Secondly, the researchers also ‘cold called’ clinics who advertised medical termination of pregnancy, or shared antenatal care. Finally, the research team was contacted by a member of a women’s health nurse union and a sexual health organisation who had both seen the project through aforementioned methods and offered to advertise through their own channels. Once a participant had been interviewed, they were also asked if they would be comfortable distributing through their own professional networks, this was added as a method after many participants offered to do so without being prompted from researchers. It was made clear to participants that they were under no obligation to do so due to the risk of their confidentiality being compromised.

### Data collection

Clinicians who showed an interest in participating were invited to undertake a semi-structured interview with Author 1, either over the phone or face to face. Interviews took place between May 2018 and September 2019. A semi-structured interview guide was developed for this study*See Additional File 1 and was utilised to allow for flexibility of discussion and answers in different settings and clinical capacities of participants and. The following questions were asked: “Can you tell me about any personal experiences that you may have had treating women who have disclosed, or you suspected, have experienced reproductive coercion?”, “What are the barriers or facilitators that you have encountered when trying to respond to reproductive coercion?”, “How do you think the current response to reproductive coercion could be improved?” and “What do you think the ideal response to reproductive coercion would look like?” The term ‘reproductive coercion’ was used in the interview context to be consistent with most of the existing literature at the time. Once all interviews were conducted, audio recordings were transcribed verbatim.

### Data analysis

Thematic analysis was the method used to analyse the data as it provided the most flexibility in exploring diverse settings, new ideas and emerging contexts within the transcripts [22]. NVivo 12 [23] was utilised to facilitate the inductive coding process laid out by Braun and Clarke [24]. This method involved an initial phase where Author 1 familiarised themselves with the data by transcribing, reading transcripts and making notes, coding the transcripts descriptively in order to group similar codes together to make interpretive codes and finally forming common themes across the dataset. Author 1 and 3 met at this point to discuss the inductive coding, authors 3 and 2 were also actively reading transcripts through this time to be familiar with the data. At this point the data was being grouped into facilitators and barriers to responding to RCA, but it became apparent that barriers made up majority of the data and these were then broken down and coded in the groups. The coding was then focused further to facilitate analysis at a deeper layer of meaning. At this point, cross-coding of a sample of transcripts was conducted by Authors 2 and 3 to ensure rigour of analysis and develop the final thematic framework of the data. This was completed over several occasions where all three authors would meet discuss the meaning behind themes and discuss subthemes/quotes where author 1 would continue to develop and discuss the themes. Once all three authors had met and cross coded on several occasions and agreed on the framework, the remainder of the transcripts were then coded using this framework. Although qualitative research is inherently subjective, the authors took steps to attempt to ensure academic rigour. All three authors brought different backgrounds (clinical health setting, feminist sociology and science) to their analysis and discussion, which helped to minimise potential bias and reach general consensus about a framework that accurately represented the data.

### Ethical considerations

The study was approved by the University of Melbourne’s Human Ethics Advisory Group (Project ID: 1853440.2). Clinicians were not offered incentives and no practice managers were advised if a clinician chose to participate. Prior to commencing any interview, informed consent was obtained from participants through reading a plain language statement and signing a consent form.

## Results

Twenty-four clinicians who worked across various settings and in different clinical roles chose to participate in this study. Clinical settings included; private general practices, subsidised community health hubs, sexual health clinics, drop-in clinics/outreach workers, GP run rural hospital and termination of pregnancy/contraception clinics. Participants were recruited from all states and territories in Australia, except the Australian Capital Territory. Majority of participants were recruited from Victoria as it was the state the researchers were based, which in turn lead to more avenues to recruit and the ability to conduct face to face interviews. Specific participant demographics can be seen below in Table 1: *Participant Demographics*. Interviews lasted on average thirty-five minutes. Two interviews were conducted as pairs; the rest of the interviews were individual. Three themes were developed which describe the barriers primary care clinicians perceived to be hindering their response to RCA: *It’s not even in the frame*, *There’s not much we can do and There’s no one to help us*. These themes were developed through a process of coding the transcripts at a descriptive, interpretive and thematic level. The themes that were the strongest in terms of number of participants expressing the same views in similar words are outlined below in detail.

**Table 1:** Participant Demographics

Variable	Number of participants
**State**	
Australian Capital Territory	0
New South Wales	4
Victoria	13
Tasmania	1
Queensland	1
Western Australia	1
South Australia	2
Northern Territory	2
	Total = 24
**Geographic Location**	
Metropolitan	10
Rural	12
Remote	2
	Total = 24
**Clinic type**	
Private general practices	4
Subsidised community health hub	6
Sexual health clinics	6
Drop-in clinics/outreach workers	2
GP run rural hospital	1
Termination of pregnancy/contraception clinic.	5
	Total = 24
**Gender**	
Female	23
Male	1
	Total = 24
**Profession**	
Nurse (includes sexual health nurse, primary care nurse)	6
Women’s health nurse	3
Doctor	15
	Total = 24

### It’s not even in the frame

Most of the clinicians acknowledged that a barrier to responding to RCA is that it is not a phenomenon that many clinicians have an awareness of. Several participants admitted their own deficiencies in this area.

*Well to be quite honest with you, I don’t think I’ve seen it [RCA], but I don’t think I’ve been keeping an eye out for it either … the fact that I don’t know very much about it or how to approach it confidently with a patient might be a barrier, on my part at least – Participant 21 (Doctor, rural)*

*Whether I've been fully aware of it or the depth of it I doubt in all cases … - Participant 14 (Doctor, urban)*

Other participants felt that they personally had a good grasp on the issue but had concerns that primary care clinicians more broadly were not aware that RCA might be an issue in primary care.

*I think probably awareness needs to be the first thing. I think [the] majority of people aren’t really aware of it, or they’re aware of it in a really vague way. – Participant 1 (Doctor, urban)*

*I think there’s a lack of awareness in this sort of general health, health profession … your average GP maybe has a slight naivete and lack of awareness of the way men use pregnancy as a controlling state … And you know I think if people aren't aware and aren't asking, they're not going to find out – Participant 6 (Doctor, rural)*

Many clinicians cited a lack of training – either formally or on-the-job – as being connected to this lack of awareness of the issue. Recognising this as a deficiency, clinicians expressed a desire to learn more around identification and responding, with training to cover both topics seen as important to provide better care to their patients. Although participants recognised this as a shortcoming in their own practice, there was no access to such training, and it was not covered in their initial medical education.

*We did have two days of a women’s health and reproductive health workshop which I think we had maybe one [day of] token domestic violence [training]. So it isn’t a huge amount – Participant 10 (Doctor, rural)*

*I don’t think it’s something that we even got taught in our general practice specialisation. There is no training, you come across some really bad consultations and situations and you have to figure out what the resources are. It should be better – Participant 12 (Doctor, urban).*

### There’s not much we can do

Participants spoke of their low levels of confidence in responding to RCA. Some clinicians suggested that even if they suspected RCA they were reluctant to ask patients about it, because they were not confident that they would respond in the “correct” manner.

*I’m trying to do the best I can for my patient, but [I’m thinking] are you doing a good job for them I don’t know … But I don’t think [I’m] confident at all – Participant 12 (Doctor, urban)*

*I think it’s one of these things that the GP does get a little bit scared of because there’s always this thing of opening a can of worms and not knowing where to go with it. – Participant 7 (Doctor, rural)*

Participants were often unsure of what services were available for women experiencing RCA and didn’t have any trusted resources for gaining this information.

*I must have to say, I do need to look it up each time … I Google domestic violence Victoria, there are some that I’ve used. It’s terrible that I’m using Google but there’s nowhere else that I can search. - Participant 12 (Doctor, urban)*

*I think it's perceived to be a very difficult topic to discuss, and people feel that they might not be able to manage the disclosure, and that they don't feel that they have the skills to be able to deal with it. I think that they don't necessarily know what services are available. – Participant 20 (Doctor, urban)*

*I'm not really up-to-date with what services we have in the state to be honest with you. – Participant 21 (Doctor, rural)*

Some clinicians were trained in screening for family and domestic violence, as well as guidelines for practice in the family and intimate partner violence context. Despite using these in practice (some because they were mandated to do so) clinicians did not think that these screening questionnaires were suited to women experiencing RCA and had reservations about their overall usefulness.

*It's one of the things we've always complained about in the screening tool [universal domestic violence screening tool]. It's been going for about 10, 12 years now and they still haven't changed it. So, we only asked about being hit, slapped or hurt, or if they're frightened to go home and if there are any children in the house. – Participant 17 (Women’s Health Nurse, rural)*

*Who are you having sex with? Is it consensual? Are you okay? Do you feel comfortable negotiating that? But again, I don't want to [ask like that]. Sometimes I do feel like it's a bit tokenistic. Kind of part of a process as opposed to exploring.” – Participant 8 (Nurse, rural)*

Many clinicians expressed frustration that although they may be trained in identifying women experiencing RCA, there wasn’t much use in doing so because there were limited services to refer women to. This was particularly an issue in rural areas, where often there were no services at all, which left clinicians on their own with nowhere to send women.

*GPs are very quick on the uptake for these things from - my experience is that if you tell us something, we'll go ‘oh right’ and start looking out for it. But if we've got nowhere to refer to or no help then it will seem like a pointless exercise … – Participant 18 (Doctor, rural)*

*I think there’s services around … like there’s not a lot of safety houses around here, there’s not services you can send people off to and say they’ll be able to look after things. You sort of have to do a bit more leg work” – Participant 4 (Doctor, rural)*

In rural and remote areas, a lack of confidentiality meant that it was not always possible to refer women to services. Often women were not willing to be referred because of the possibility of their situation being made worse by other community members becoming aware.

*The tricky bit with services in your own town in rural areas is the issue of confidentiality. People are fearful that the people they know work in the services and therefore people are gonna know what they're accessing or their issues that come with that. – Participant 11 (Nurse, rural)*

*The problem is that when they travel [to get an abortion] everyone knows. - Participant 18 (Doctor, rural)*

*In a remote setting it’s another thing again because you’ve got limited number of health workers, a lack of privacy within the community. – Participant 3 (Doctor, Rural)*

### There’s no one to help us

Many clinicians found themselves in a difficult position when attempting to refer women experiencing RCA to other services. Some clinicians spoke about how they were unable to refer women they identified as experiencing RCA because services wouldn’t accept these women. At times, participants felt that specialist domestic violence services did not recognise the level of risk associated with RCA, or did not perceive RCA as a form of domestic violence.

*What is important to me, what I am seeing as risk doesn’t necessarily marry with what they [domestic violence service] deem as risk. So there have been cases where I have reported things and there has been no action and I have been at a loss to understand why. – Participant 19 (Nurse, urban)*

*Women talking about reproductive coercion, I imagine a fair percentage of those wouldn’t see it as domestic violence ... To call a DV helpline, and I again, I have no idea, but I’m just kind of taking a punt that staff at the end of the line, what experience would they have in talking around pregnancy and contraception would be limited- Participant 22 (Women’s Health Nurse, urban)*

Further complicating the disconnect between RCA and domestic violence services was the overall lack of ‘space’ in programs. Services weren’t taking new clients, or prioritised women experiencing severe physical violence. This was a challenge for clinicians when responding to RCA, across both urban and rural areas.

*If they [women] did want to go through our Family Violence program, they're not taking any new clients at the moment – Participant 14 (Doctor, rural)*

*The issue with the services we have is that we just do not have enough refuge beds on a daily basis … which is kind of the tip of ice of the iceberg stuff. So, if we can't even manage the really tip of the iceberg, then I think the others, we really do need to focus on more service provision at the very, very basic primary care level. – Participant 20 (Doctor, urban)*

Responding to women experiencing pregnancy coercion or pressure in a timely manner was emphasised as being a concern. Unlike identifying and responding to other forms of intimate partner violence, RCA often has a small window of time within which women must receive a response before a pregnancy will have progressed too far for clinicians to be able to offer women choices. Clinicians expressed the difficulties they encountered in trying to bring in other services to help support patients when these services could not connect within medical timeframes.

*They can um they can see psychologists, but you know there’s often costs and things involved and um if they’re wanting a medical termination then they’ve got a fairly finite time that they can make the decision in. - Participant 2 (Doctor, urban)*

*Timing is pretty crucial as I said, the longer the pregnancy goes on, the more complicated the abortion might be and also it might not be an option because a surgical termination might be too expensive and there’s more out of pocket expenses. – Participant 24 (Women’s Health Nurse, urban)*

*The other problem is if you do pick it up then refer off to services but in the [referral] service have their own waiting times and trying to get things in place [is difficult]. – Participant 10 (Doctor, rural)*

When asked what barriers they experienced specific to their area in Australia, clinicians highlighted the negative impact laws around abortion in each state had on their ability to either provide or facilitate access to abortion for their patients. Clinicians spoke of the negative effect that high costs of abortion had on women, meaning that some women were forced to continue pregnancies that they didn’t want.

*If that person then decides that the pregnancy continuation is the only option because they can't afford $1200 to go two hours’ drive to get this termination - and even to get the medical termination, they've still got to drive, get the pills, and then they've got to go back in two weeks for an ultrasound. So that's a system thing. – Participant 17 (Women’s Health Nurse, Rural)*

*For medical abortions, in South Australia because termination has to occur in a hospital setting, that means that medication abortion has to occur in the hospital, so they have to be given the tablets in the hospital. Which, you know, you've got your country areas that it's just not feasible - Participant 19 (Nurse, Urban)*

*I think probably just access to abortion is a terrible barrier in NSW of if you do need to get someone quickly somewhere, or quickly through some clinics, that’s a terrible barrier for us. That that process often ends up in negotiating the abortion provider and the social worker as well. It ends up taking more time and that ends up having more risk for that person as well. - Participant 23 (Nurse, urban)*

## Discussion

This study highlighted several barriers to responding to RCA in Australian primary care, both directly from the clinician themselves and more systemically. Clinicians were insightful in evaluating their own practice and how they acted as a barrier themselves through their own lack of awareness. This is the first study that investigated clinicians experiences in primary care, however a lack of awareness around RCA by clinicians and the community has been identified as an issue in Australia [3]. Previous studies in Australia were conducted in a tertiary hospital setting, where the challenges for clinicians centred around a lack of shared understanding between different clinicians and the need for a multidisciplinary approach [3, 20]. The clinicians in the hospital did not face any barriers to access any services like the clinicians in primary care do, as services are readily available and co-located within the hospital. Although it would improve barriers by having services co-located with primary care clinics, in the current climate, this would not be logistically and financially viable. Further research is needed to investigate how to better streamline access to services from primary care, to bridge the gap between services and improve outcomes for women.

Guidelines for best practice are yet to be formulated in the Australian context, but comparing the current Australian literature with this study shows that guidelines can’t be a ‘one size fits all’ like current guidelines in the US [25] as the barriers and access to services in Australia are so diverse.

Currently there is no evidence in the extant literature supporting the use of screening questions for RCA, or what guidelines should be recommended in Australia, however, the American College of Obstetricians and Gynecologists [26] and the United States organisation Futures Without Violence [25] have both published guidelines for responding in the US context. Although these guidelines are similar to the World Health Organisation recommends for addressing intimate partner violence (such as the need for ‘woman centred care’) [9], these US guidelines simply do not translate into the Australian healthcare system. These guidelines rely on a system that is specialised to sexual health and gynaecological care, which is not the case in Australia where a major portion of sexual and reproductive health occurs in primary care, rather than specialist centres. If proven to be effective, future research could focus on working with clinicians, stake holders and government health bodies to develop screening in the Australian health context, that would encompass the different settings found in primary care.

Many of the clinicians in this study felt disheartened and exasperated when trying to assist their patients. Although they identified RCA, often there was nowhere to refer women for further assistance, especially in rural areas. This is an extremely serious barrier in terms of women’s safety, given that we know that women who experience RCA are more often than not experiencing other forms of abuse [2, 5]. Although this issue was experienced across different locations, it was of more concern to clinicians working rurally. It was emphasised that women living rurally have less access to health services overall. This is consistent with previous literature that shows women living rurally are more disadvantaged when it comes to access to healthcare [15, 27, 28].

Regardless of location, many clinicians described how domestic violence services often did not recognise the risk associated with reproductive coercion and abuse. Future research needs to focus on domestic and family violence services and how they respond to RCA in Australia. This could lead to identifying deficits in knowledge and educating these providers in the same way clinicians are around negative health impacts and potential red flags for further abuse. It may be that while this form of violence has become more recognised in the health context due to the nature of the necessity for treatment, it is not recognised in the same way in these services. This could also help to make improvements to the current referral pathways from healthcare into domestic violence services.

A key finding of this study is the incompatibility between the numerous barriers to identification and response in primary care, problems around safe access to abortion, and the time-sensitive nature of an unwanted pregnancy, particularly for rural women. Literature shows that there are barriers to access abortion in primary care in rural areas as GPs in the area are conscientious objectors to abortion [29]. For women experiencing RCA, accessing medical termination of pregnancy medication is important as they can take this in the safety of their home, which in some cases may be the safest option. As the law in Australia requires women to access to medical abortion only up to nine weeks gestation [14], women experiencing RCA may present outside that window, leaving them at greater risk. Further emphasis on ensuring there are adequate numbers of doctors trained to prescribe termination of pregnancy medication could potentially be a focus for local primary health networks to address the lack of providers in rural areas.

### Limitations

Although there were strengths to this study in that clinicians were recruited from very diverse settings, it is also important to acknowledge the limitations. Most significantly is the use of purposive sampling during recruitment, where clinicians who were more likely to have experience in their respective fields participated, meaning that their response to interview questions may not accurately represent the general population of primary care clinicians. There is also the potential for bias where majority of participants were from Victoria, there is a possibility that not all barriers from other states have been explored. Only one participant was male, which although this reflects the primary care nurse demographic, it does not reflect the population of general practitioners [30]. The final limitation is the lack of antenatal providing clinicians recruited, although the research team took steps to specifically recruit antenatal shared care GPs, no GPs from this group were recruited.

## Conclusions

Despite increased recognition in research and practice [3, 20, 31], the results show that there is a lot more work needs to be done to address the barriers to responding to RCA in Australian primary care. Although clinicians are willing and able to be trained to identify and respond to reproductive coercion and abuse, in the current system there would be little benefit if there is nowhere to refer women. Strategies need to be undertaken to make the referral process from healthcare to domestic violence/counselling services easier for all parties in involved. More needs to be done at a policy level to improve access to abortion, such as telehealth for women living rurally to minimise delay in access, cost of travel and greater privacy and access through the public health system where currently only private is available and unreachable for many women. Further research needs to focus on referral services after RCA is identified in healthcare, to understand where the current breakdowns in the process exist.

## Supplementary Information

**Additional file 1.** Semi-structured interview guide. List of interview questions guiding the semi-structured interview.

## Data Availability

Due to the nature of this research, participants of this study did not agree for their data to be shared publicly, as per approved ethics application so supporting data is not available.

## Abbreviations

RCA: Reproductive coercion and abuse
IPV: Intimate partner violence
SV: Sexual Violence
WHO: World Health Organisation
GP: General Practitioner
PHN: Primary Health Network

## References

[1] Grace KT, Anderson JC. Reproductive coercion: a systematic review. Trauma Violence Abuse. Futures Without Violence; 2018;19(4):371–90. 10.1177/1524838016663935. 10.1177/1524838016663935 PMC5577387 27535921

[2] Miller E, Jordan B, Levenson R, Silverman JG (2010). Reproductive coercion: connecting the dots between partner violence and unintended pregnancy. Contraception..

[3] Tarzia L, Wellington M, Marino J, Hegarty K. “A huge, hidden problem”: Australian health practitioners’ views and understandings of reproductive coercion. Qual Health Res. 2019;29(10):1395–407. 10.1177/1049732318819839 30584793

[4] Grace KT, Fleming C (2016). A systematic review of reproductive coercion in international settings. World Med Heal Policy.

[5] Sutherland MA, Fantasia HC, Fontenot H (2015). Reproductive coercion and partner violence among college women. JOGNN.

[6] McCauley HL, Falb KL, Streich-Tilles T, Kpebo D, Gupta J (2014). Mental health impacts of reproductive coercion among women in Côte d’Ivoire. Int J Gynecol Obstet.

[7] Northridge JL, Silver EJ, Talib HJ, Coupey SM (2017). Reproductive coercion in high school-aged girls: associations with reproductive health risk and intimate partner violence. J Pediatr Adolesc Gynecol.

[8] Coker AL (2007). Does physical intimate partner violence affect sexual health? A systematic review. Trauma Violence Abuse.

[9] World Health Organisation. Health care for women subjected to intimate partner violence or sexual violence, A clinical handbook. Geneva: World Health Organistation; 2014.

[10] García-Moreno C, Hegarty K, d’Oliveira AFL, Koziol-McLain J, Colombini M, Feder G (2015). The health-systems response to violence against women. Lancet (London, England).

[11] World Health Organisation. Responding to intimate partner violence and sexual violence against women: WHO clinical and policy guidelines [Internet]. Geneva: World Health Organisation; 2013. [cited 2020 Jul 28]. Available from: https://www.who.int/reproductivehealth/publications/violence/9789241548595/en/. 24354041

[12] Australian Government Department of Health. Primary care [Internet]. Canberra: Commonwealth of Australia; 2020 [updated 2020; cited 2020 Jul 28]. Available from: https://www1.health.gov.au/internet/main/publishing.nsf/Content/primarycare

[13] Australian Government Department of Health. About the GP Super Clinics Programme [Internet]. Canberra: Commonwealth of Australia; 2013 [updated 2020; cited 2020 Jul28]. Available from: https://www1.health.gov.au/internet/main/publishing.nsf/Content/pacd-gpsuperclinic-about

[14] Mazza D, Burton G, Wilson S, Boulton E, Fairweather J, Black K (2020). Medical abortion. Aust J Gen Pract [Internet].

[15] Doran FM, Hornibrook J (2016). Barriers around access to abortion experienced by rural women in New South Wales, Australia. Rural Remote Health.

[16] de Moel-Mandel C, Shelley JM (2017). The legal and non-legal barriers to abortion access in Australia: a review of the evidence. Eur J Contracept Reprod Heal Care [Internet].

[17] Feder GS, Hutson M, Ramsay J, Taket AR (2006). Women exposed to intimate partner violence: expectations and experiences when they encounter health care professionals: a meta-analysis of qualitative studies. Arch Intern Med.

[18] Yeung H, Chowdhury N, Malpass A, Feder GS (2012). Responding to domestic violence in general practice: a qualitative study on perceptions and experiences. Int J Family Med [Internet].

[19] Cox P (2016). Violence against women: additional analysis of the Australian Bureau of Statistics’ personal safety survey, 2012. 01.01/2016.

[20] Tarzia L, Wellington M, Marino J, Hegarty K. How do health practitioners in a large Australian public hospital identify and respond to reproductive abuse? A qualitative study. Aust N Z J Public Health. 2019;43(5):457–63. 10.1111/1753-6405.12923 31268221

[21] Australian Government Department of Health. The Australian health system [Internet]. Canberra: Commonwealth of Australia; 2019 [updated 2019, cited 2020 Jul 28]. Available from: https://www.health.gov.au/about-us/the-australian-health-system

[22] Nowell LS, Norris JM, White DE, Moules NJ. Thematic Analysis: Striving to Meet the Trustworthiness Criteria. Int J Qual Methods. 2017;16(1).

[23] NVIVO: qualitative data analysis software. Version 12. Doncaster (AUST): QSR International; 2018.

[24] Braun V, Clarke V (2006). Using thematic analysis in psychology. Qual Res Psychol [Internet].

[25] Chamberlain L, Levenson RR. Addressing intimate partner violence reproductive and sexual coercion: a guide for obstetric, gynecologic, Reproductive Health Care Settings. San Francisco; 2013.

[26] American College of Obstetricians and Gynecologists (2013). Committee Opinion No. 554: reproductive and sexual coercion. Obstet Gynecol.

[27] Hooker L, Theobald J, Anderson K, Billet P, Baron P (2019). Violence against young women in non-urban areas of australia: a scoping review. Trauma Violence Abus [Internet].

[28] Dobson A, Byles J, Dolja-Gore X, Fitzgerald D. Rural, remote and regional differences in womens health: findings from the Australian longitudinal study on womens health [internet]. Women's Health Australia; 2011. Available from: https://ranzcog.edu.au/RANZCOG_SITE/media/RANZCOG-MEDIA/About/NWHS/Resources/Rural,-remote-and-regional-differences-in-womens-health.pdf.

[29] Keogh LA, Gillam L, Bismark M, McNamee K, Webster A, Bayly C, et al. Conscientious objection to abortion, the law and its implementation in Victoria, Australia: perspectives of abortion service providers. BMC Med Ethics [internet]. 2019;20(1):11. Available from: 10.1186/s12910-019-0346-1. 10.1186/s12910-019-0346-1 PMC6354355 30700292

[30] Medical Board of Australia. Medical Board of Australia Registrant Data [Internet]. 2020.

[31] Price E, Sharman LS, Douglas HA, Sheeran N, Dingle GA. Experiences of reproductive coercion in queensland women. J Interpers Violence. 2019. In Press. 10.1177/0886260519846851 31057040

