# Supplementary material for: Barriers to responding to reproductive coercion and abuse among women presenting to Australian primary care
Source: BMC Health Serv Res. 2021 May 4;21:424. doi: 10.1186/s12913-021-06420-5 (PMC8097864; doi:10.1186/s12913-021-06420-5)
Supplement: Supplementary file 1 — Additional file 1. Semi-structured interview guide. List of interview questions guiding the semi-structured interview. [file 12913_2021_6420_MOESM1_ESM.pdf]

Additional File 1: Semi-structured interview guide

1. What does the term reproductive coercion mean to you?

2. How often roughly would you see this in your practice?

3. Can you describe a time when one of your patients was experiencing RC?

4. How would you identify that a patient may be experiencing RC?

5. What did you do when it became known that a patient was experiencing RC?

6. How confident do you feel in responding?

7. What are the barriers and facilitators to responding?

8. What do you think is best practice?

9. How do you think the response could be improved?
